# Strategies to prevent workplace sexual harassment among Iranian nurses: A qualitative study

**DOI:** 10.3389/fpsyg.2022.912225

**Published:** 2022-08-22

**Authors:** Maryam Zeighami, Mohammad Ali Zakeri, Parvin Mangolian Shahrbabaki, Mahlagha Dehghan

**Affiliations:** ^1^Department of Nursing and Midwifery, Kerman Branch, Islamic Azad University, Kerman, Iran; ^2^Nursing Research Center, Kerman University of Medical Sciences, Kerman, Iran; ^3^Determinants of Health Research Centre, Non-communicable Diseases Research Center, Rafsanjan University of Medical Sciences, Rafsanjan, Iran; ^4^Department of Critical Care Nursing, Razi Faculty of Nursing and Midwifery, Kerman University of Medical Sciences, Kerman, Iran

**Keywords:** strategy, workplace, sexual harassment, nurses, qualitative study

## Abstract

**Background:**

Sexual harassment in the workplace has many negative consequences for nurses and the delivery of patient care. Appropriate policies and strategies can help to create a safe work environment for nurses. Therefore, the present study aimed to investigate Iranian nurses’ strategies for preventing sexual harassment in the workplace.

**Materials and methods:**

This qualitative descriptive-explorative study used conventional content analysis to investigate how Iranian nurses cope with sexual harassment (*n* = 22). Participants were selected using a purposeful sampling method. Data was collected through in-depth, semi-structured interviews from September 2020 to April 2021. In order to obtain rich information, maximum variation was considered (age, sex, work experience, level of education, marital status, and type of hospital and ward). The Guba and Lincoln criteria were used to increase the study’s trustworthiness, while the Graneheim and Lundman approach was used to analyze the content.

**Results:**

One hundred and twelve codes, one main category, four categories, and 12 subcategories were extracted. The main category, strategies to prevent sexual harassment among nurses in the workplace, includes four categories: behavioral response, working conditions adjustment, informing, and performance of hospital security guards. The most common strategy used by nurses was behavioral response.

**Conclusion:**

Basic measures are required to prevent sexual misconduct against nurses, which is an obvious part of the professional organizational culture. Managers and policymakers should develop workplace ethics, legal accountability, and safety. They should also develop training programs and prevention strategies to help nurses improve their coping skills. Further quantitative and qualitative research in other healthcare groups is required to confirm the findings of this study.

## Introduction

Sexual harassment in the nursing system is an acute condition that has been experienced by some nurses in various ways in the workplace (Zeighami et al., 2022). Unwelcome sexual advances, behaviors, and other verbal or physical conduct of a sexual nature constitute sexual harassment when this conduct violates an individual’s dignity and creates an intimidating, hostile, degrading, humiliating, or offensive environment (NFA, 2016; Danielsen and Dfaapa, 2018). Sexual harassment in the nursing system is divided into five categories: verbal, physical, visual, seductive, and cyberbullying. However, patients and their families, doctors, colleagues, and other hospital staff may harass a nurse (Zeighami et al., 2022). According to a survey of Danish employees, the prevalence of sexual harassment was 3.1% across all occupations, but 16.4% among healthcare workers (NFA, 2016).

Ten to 87% of nurses reported sexual harassment (Berry et al., 2012; Spector et al., 2014; Kahsay et al., 2020). According to Zeighami et al. (2021), nurses were sexually harassed in the workplace in a variety of ways (Zeighami et al., 2022), which had negative mental health consequences or short-term and long-term problems for nurses (Friborg et al., 2017). Sexual harassment has increasingly affected the health and safety of nurses as well as the quality and efficiency of healthcare systems (Adams et al., 2019). Sexual harassment has a number of negative consequences, including psychological disorders, detrimental occupational effects, physical injury, and a lack of warm family relationships, all of which have a negative impact on their personal and professional lives and place a heavy burden on the healthcare system due to decreased productivity and loss of active labor (Zeighami et al., 2021). Several studies have addressed the negative psychological effects of sexual harassment on nurses, for example the investigations conducted in Denmark (Nielsen et al., 2017), South Korea (Kim et al., 2018), Sub-Saharan Africa (Tollstern Landin et al., 2020), and Iran (Zeighami et al., 2022). Sexual harassment can cause a sense of discomfort, shame (Tollstern Landin et al., 2020), severe negative emotions, fear, and mental imbalance (Nielsen et al., 2017; Kim et al., 2018) as well as mental health disorders (Nielsen et al., 2010; Ali and Ezz El Rigal, 2019) among nurses. Sexually harassed nurses are unable to do the right thing and have a poor quality of life in a frightening, hostile, or offensive work environment (Bondestam and Lundqvist, 2020). Sexual harassment reduces proper patient care (Nielsen et al., 2017; Kim et al., 2018). Ali and Ezz El Rigal (2019) showed that sexual harassment was prevalent in clinical settings, which resulted in poor practice and burnout of nurses (Ali and Ezz El Rigal, 2019).

Studies have shown a variety of reasons for sexual harassment in the workplace. Both individual (such as age, women, level of education, marital status, and minorities) and organizational (organizational climate, job-gender context, and relative power between the harasser and the victim) factors predict sexual harassment (Siuta and Bergman, 2019). Organizational climates that are more tolerant of sexual harassment produce more sexual harassment. Those with lower organizational power are more likely to experience sexual harassment (Siuta and Bergman, 2019). In addition, organizations might have overlooked sexual harassment (Krøjer et al., 2014). Nielsen et al. (2017) found that there were few guidelines or policies in the workplace to manage and/or prevent sexual harassment (Nielsen et al., 2017).

However, factors such as poor working conditions, hierarchical organizations, the normalization of gender-based violence, cultural context, and a lack of active leadership have all contributed to sexual harassment (Bondestam and Lundqvist, 2020). Therefore, managers and policymakers must develop guidelines for legal accountability and safety at work, as well as training programs for nurses to improve their coping skills (Zeighami et al., 2022). Appropriate workplace policies and strategies can help to create a safe environment for nurses (NFA, 2016). Basic measures must be taken to manage and deal with sexual harassment, which is an obvious part of the problem of organizational-professional culture (Nielsen et al., 2017). Therefore, extensive knowledge of Iranian nurses’ coping strategies with sexual harassment in the workplace will provide adequate support to victims of sexual harassment (Zeighami et al., 2021). The experiences of nurses can help to develop the necessary interventions to identify coping strategies and reduce sexual harassment in the workplace (Najafi et al., 2018).

Understanding workplace violence experiences encourages nurses, organizations, and professional centers to prepare nurses for workplace violence and to implement appropriate interventions to reduce the occurrence of this ominous social phenomenon (Kvas and Seljak, 2014). Given the sensitivity of sexual harassment, it is important to understand how nurses view it. Nurses’ opinions, given the experiences that have closely touched on these problems, can be the best way to recognize sexual harassment and support nurses in responding to sexual violence in the workplace. Therefore, effective approaches and methods such as qualitative studies should be used to identify the problem of sexual harassment so that nurses can express their experiences to help better solve the problem. This qualitative study aimed to investigate Iranian nurses’ strategies for preventing sexual harassment in the workplace. Nurse managers can use the insights presented in this study to identify coping strategies and reduce sexual harassment among nurses.

## Materials and methods

### Study design and setting

This qualitative descriptive-explorative study was conducted by using conventional content analysis, a research approach for the description and interpretation of the textual data, as well as the identification of implicit and explicit themes using a systematic process of coding (Hsieh and Shannon, 2005). According to Graneheim and Lundman (2004) and Thyme et al. (2013), each text contains implicit and explicit messages that, while they must be interpreted, vary in depth and level of abstraction. We tried to analyze the implicit and explicit content. This study was conducted in Kerman, the largest city in southeastern Iran.

### Sampling, participant, and data collection

The sampling method was based on purposive sampling. We first approached the relevant authorities at the hospitals and asked them to report any cases of sexual harassment that they were tendency to participate in the interview. Some of the samples were also selected as snowballs, which were introduced by the participants. In this study, we tried to select participants who had rich experiences with the phenomenon. Maximum variation sampling was used to select participants. Female and male nurses working in different ward of hospitals affiliated with Kerman University of Medical Sciences (KUMS) as well as those working in private hospitals were interviewed. As there is no rule to determine the number of participants in a qualitative study, our sampling continued until data saturation and the extraction of no new information (Mansourian et al., 2019). In the present study, after 18 interviews, the data appeared to be saturated. Therefore, four more interviews were conducted to ensure data saturation. After coding and analyzing the data, no new information was added to the existing data. Thus, data saturation was obtained. In addition, 18 females and four males participated in the study. The major Iranian nurses particularly in our setting are females. In the study setting nearly 85% of the nurses are female. The interviews were conducted with pre-arranged appointments with participants. The location of some of the interviews was chosen by the nurses in the hospital lounge. In some cases, a nursing school or coffee shop was chosen as the interview location. In all cases, we tried to choose a quiet and secluded environment for the interview. Nurses with diverse and rich experiences were interviewed to obtain rich and varied information. Various personal and occupational characteristics such as age, marital status, level of education, work experience, position, type of hospital (public, private, and educational) and ward were selected to provide a wide range of information. Eighteen female and four male nurses participated in the study. They were between 25 and 51 years old and had 2–28 years of work experience (Table 1). Inclusion criteria included nurses working in hospitals affiliated with Kerman University of Medical Sciences and private hospitals who had a bachelor’s degree or higher and had at least 1 year of clinical experience. Other inclusion criteria were a history of sexual harassment in the workplace and a tendency to participate in interviews. Exclusion criteria included people with a history of mental illness or a history of alcohol or drug addiction. The first researcher conducted semi-structured and face-to-face individual interviews. Table 2 lists some of the questions that reflect participants’ experiences with strategies to cope with sexual harassment. The interviews lasted between 30 and 100 mins, and they were recorded and transcribed verbatim. Sampling was performed from September 2020 to April 2021.

**TABLE 1:** Characteristics of participants (*n* = 22).

Participants		
Sex	Female	18
	Male	4
Age (year)	Minimum	25
	Maximum	51
Marital status	Single	7
	Married	12
	Divorced	3
Work experience (year)	Minimum	2
	Maximum	28
Education level	Bachelor’s	15
	Master’s	6
	Ph.D.	1
Employment	Contract recruiters-1^a^	4
	Contract recruiters-2^b^	5
	Hired	13
Position	Nurse	14
	Head nurse	2
	Supervisor	3
	Faculty member	3
Type of hospital*	Public-educational	20
	Public-non-educational	1
	Private	9
Type of ward*	Emergency	11
	Medical	8
	ICU	7
	Operating room	6
	CCU	5
	General surgery	4
	Nursing management department	3
	Orthopedics	3
	Psychiatrics	3
	Pediatrics	2
	Digestive	2
	Burn	1
	Dialysis	1

*Some participants had work experience in different hospitals and ward.

^a^Annually contracted with payment less than hired nurses.

^b^Annually contracted with payment similar to hired nurses.

**TABLE 2:** Example of questions.

Questions
1- Would you share your experience of sexual harassment at work?
2- How did you react to sexual harassment?
3- What strategies have you used to prevent such harassment?

### Data analysis

The Graneheim and Lundman methods were used for content analysis. At the same time as collecting information, the data analysis process was performed. Recorded interviews were transcribed. Due to the fact that in qualitative research it is necessary for the researcher to be immersed in the data, the interviews were read several times to get a general sense of the text of the interview. In this study, each interview was considered as a unit of analysis. Then, the analysis units were divided into meaning units. After summarizing and condensing of meaning units, coding was performed. The codes were divided according to similarities and differences under different categories and subcategories. The meaning units were condensed and then coded, and subcategories and categories were created. The main category in the present study was strategies to prevent sexual harassment. Table 3 provides an overview of the data analysis process, and Table 4 provides an overview of all categories and subcategories. The analysis process was performed from September 2020 to November 2021.

**TABLE 3:** Example of qualitative content analysis process.

Meaning unit	Condensation	Code	Subcategories	Categories	Main category
I was caring for an ill patient who had been hospitalized for some time, and I treated him with kindness. When he recovered, he proposed a love affair with me. It was my fault for failing to observe the boundaries, and thus he permitted himself to make this offer. I should have avoided using terms like “my dear”. Privacy must be respected to prevent these occurrences	Kindness and intimacy with the patient and non-observance of boundaries and privacy and the patient’s misconception and offer to establish a love relationship with a nurse	Observing privacy with patients as a deterrent factor of sexual harassment	Communications privacy	Behavioral response	Strategies to prevent sexual harassment
A male coworker sent me a romantic message. We were both married. During my night shifts, he discussed sex with me. I asked the head nurse not to schedule me on the same shift as him. This is how I got rid of him	A married male nurse harassed a married female nurse by sending love messages and engaging in sex talk	Separating and controlling harassers as a strategy to prevent sexual harassment	Management of scheduling	Working conditions adjustment	
I was alone in the examination room. Then, one of the male patients came over and hugged me. As a novice, I did not know I had to be careful. I even kept an eye on what was going on behind me. Nobody told me to be careful when I was in a situation like this	Male patients’ harassment of female nurses as a result of a lack of nurse’s education on such harassment	Educating and warning nursing staff about sexual harassment in the workplace	In-service training to reduce sexual harassment among staff	Informing	
I once spent the night alone in a nursing station. The escort of the patient approached and attempted to harass me. I pointed to the camera. Interestingly, he took a step back immediately upon noticing the camera	The patient’s escort approached the nurse to harass her, but backed away when he saw the camera	Installing cameras in hospitals to prevent sexual harassment	Installing a camera to prevent sexual harassment	Hospital security guards	

**TABLE 4:** Subcategories, categories, and main category extracted from qualitative content analysis.

Subcategories	Categories	Main category
- Deterrent behaviors - Communications privacy - Appropriate work attire	Behavioral response	Strategies to prevent sexual harassment
- Change of ward - Change of shift work - Management of scheduling	Working conditions adjustment	
- Academic education on sexual harassment - In-service training to reduce sexual harassment of staff - Education on sexual harassment in childhood	Informing	
- Legal action against the harasser - Installation of a camera - Rapid tracking of sexual harassment reports	Performance of hospital security guards	

### Trustworthiness

The study’s trustworthiness was evaluated based on four criteria proposed by Lincolon and Guba, including credibility, confirmability, dependability, and transferability (Kyngas et al., 2020). For data credibility, the researcher attempted to collect and analyze data for a long time (more than a year) while maintaining a positive relationship with the participants. She also attempted to collect detailed data by using a variety of samples. Following the analysis of each interview, member checking was used to clarify ambiguities and confirm the extracted ideas. Furthermore, two experienced researchers analyzed and interpreted the data, and the research team compared the data and reviewed codes to increase credibility. During the research process, the research team created a mind map to confirm the data. Peer checking was used to examine the transcripts of interviews, codes, categories, and data coding processes. External checkers were used to examine similarities in perception between them and the researcher and to seek differences. To increase the transferability, the research findings were given to two samples who were not participants, and their opinions were used to develop a conceptual generalization.

### Findings

The qualitative content analysis explained and defined the meaning, dimensions, and components of strategies to prevent sexual harassment in the workplace. The findings of this study were in the form of a main category, four categories, and 12 subcategories. In total, 112 codes remained after continuous comparative analysis, code condensation, and integration (Table 4). Strategies to prevent sexual harassment in the workplace were explained based on participants’ experiences.

### Main category: Strategies to prevent sexual harassment in the workplace

According to participants, the strategies to prevent sexual harassment in the workplace include four categories of behavioral response, working conditions adjustment, informing, and performance of hospital security guards.

#### Behavioral response

Behavior is a reaction to an action. A person can convey a concept without saying a word and only with body language. This issue arises from the processes that took place in his mind. Behavioral reactions are the coping styles that people display in dangerous situations. Nurses participating in the study believed that behavioral responses were among the strategies to prevent sexual harassment in the workplace that were in the form of deterrent behaviors, communications privacy, and appropriate work attire.

##### Deterrent behaviors

The majority of participants indicated that developing deterrent behaviors was one strategy for preventing workplace sexual harassment. These behaviors included failing to listen, ignoring, acting harshly, mistreating, and frowning at the harasser.

*“When my male coworker and I were alone at the station, he began talking about sex, but I tried not to listen. I was afraid that if I said anything, things would get worse” (Participant No. 12, a female nurse with 5 years of clinical experience).*

*“I had to perform an ECG on a young female patient in the emergency room. I connected the electrodes without touching any of her buttons. Suddenly, she started to open her buttons, exposing her chest on purpose. I had a bad feeling and tried not to look at her chest” (Participant No. 21, a male nurse with 5 years of clinical work experience).*

##### Communications privacy

Some nurses in the study argued that keeping a safe distance, avoiding getting too friendly, not making jokes, and respecting the privacy of patients, doctors, and staff were all effective ways to prevent sexual harassment.

*“I had a great deal of respect for physicians and was extremely friendly with them. One of the doctors misinterpreted my behavior and did not maintain a safe distance from me. I realized that the only thing that mattered was behavior, and that there was no distinction between a physician and a layperson in this regard. As a result, I attempted to keep a safe distance” (Participant No. 8, a 25-year-old female nurse with 4 years of clinical work experience).*

*“I did not anticipate encountering these issues in the workplace when I began my career. I once paid several visits to a female patient in a private room, where we joked and laughed together. The patient misinterpreted my behavior, took my hand, and proposed an affair to me. As a result, I tried to maintain my distance and privacy” (Participant No. 20, a 28-year-old male nurse with 7 years of clinical work experience).*

##### Appropriate work attire

According to some male and female nurses who participated in the study, refraining from wearing tight clothing and using cosmetics could be a strategy for preventing workplace sexual harassment.

*“It is critical to wear appropriate work attire because it determines everyone’s body language, dignity, and personality. Female nurses who used cosmetics and wore tight clothing were more likely to be harassed” (Participant No. 11, a male nurse with 5 years of clinical work experience).*

#### Working conditions adjustment

According to nurses, this issue should be managed in such a way as to avoid being in a situation that may lead to sexual harassment. Nursing managers must identify and moderate the factors that lead to sexual harassment. Nurses participating in the study believed that working conditions adjustment could reduce or prevent sexual harassment in the forms of change of ward, change of shift work, management of scheduling.

##### Change of ward

The majority of study participants reported that changing their work environment made them less likely to face the harasser and, on the other hand, caused them to leave the environment where they were harassed in order to forget about sexual harassment.

*“When one of the doctors saw me in a secluded area, he began harassing me, and touching my body. I did not do anything wrong to provoke him, but he did not give up. To get rid of him and maintain my dignity, I changed the ward where I was working” (Participant No. 17, a female nurse with 19 years of clinical work experience).*

##### Change of shift work

According to study participants, the majority of sexual harassment incidents occurred during night shifts, particularly in ward with young male patients and critical care units. Managing night shift work might thus be effective in reducing sexual harassment.

*“I was harassed mostly at night shifts, and working at night shifts caused me a lot of stress. I was afraid of my own shadow. I requested they not schedule any night shifts for me” (Participant No. 4, a female nurse with 26 years of clinical work experience).*

##### Management of scheduling

The study participants believed that if female nurses cared for female patients and male nurses cared for male patients, sexual harassment would be less likely to occur, or that a combination of young and experienced staff on each shift would prevent sexual harassment. Additionally, personnel should be organized in such a way that doctors and other personnel cannot sexually harass nurses, particularly on night shifts.

*“I was a nurse in the men’s orthopedic ward. Most of the patients were young. Sometimes, when I went into their room, they would make fun of me. This made me enter their room with a sense of dread and reluctance. They touched me several times. They should have a male nurse” (Participant No. 9, a female nurse with 4 years of clinical work experience).*

#### Informing

Informing is essential for understanding the correct and practical methods of dealing with sexual harassment and is done through continuous training. Education is an endless process. Many nurses participating in the study had no idea how to respond or behave in the face of harassment. Academic education on sexual harassment, in-service training to reduce staff sexual harassment, and education on sexual harassment in childhood are all ways to get information about sexual harassment.

##### Academic education on sexual harassment

Nurses participating in the study argued that they should be trained on how to cope with sexual harassment. They received no training in this area during their studies and could not discuss it due to the taboo surrounding sexual harassment.

*“I was caring for a male patient when he abruptly exposed his genital organ. I was taken aback and did not know what to say. I had to be taught what to do in these situations” (Participant No. 3, a female nurse with 23 years of clinical work experience).*

##### In-service training to reduce sexual harassment of staff

The study participants suggested that in-service training of sexual harassment could be an effective way to cope with sexual harassment. Nursing staff should be trained on how to react in such situations and who to refer to for guidance.

*“When I was harassed by a doctor, my mind became extremely busy. Instead of reporting it, I replayed it through my head and killed that doctor a thousand times. I always wish I could have done something. Nurses should be educated and reminded of their rights in such situations, and they should not remain silent” (Participant No. 6, a female nurse with 2 years of clinical work experience).*

##### Education on sexual harassment in childhood

Some study participants believe that education on sexual harassment should begin in childhood. The child should understand what sexual harassment is and how to deal with it.

*“Training should start in school. Children should know what sexual harassment is, and what to do if it occurs, which will increase their self-esteem” (Participant No. 2, a female nurse with 26 years of clinical and educational work experience).*

#### Performance of hospital security guards

According to the nurses who participated in the study, taking legal action against the harasser, setting up a camera, and quickly tracking sexual harassment reports were all good ways to deal with workplace sexual harassment.

##### Legal action against the harasser

Some nurses reported that when they were sexually harassed, the security guards handled the situation effectively.

*“I was injecting the patient’s medicine when the escort of the patient touched me. I called the security guard. They took the abuser out of the ward and did not allow him to go to the patient’s bedside.” (Participant No. 10 female nurse with 8 years of clinical experience).*

##### Installation of a camera

According to some nurses participating in the study, the installation of cameras in different places of the hospital was effective.

*“Once I was in the elevator, the doctor touched me. Because he knew there was no camera in the elevator” (Participant No. 1, a female nurse with 2 years of clinical work experience).*

##### Rapid tracking of sexual harassment reports

Some nurses participating in the study suggested that prioritizing reports of sexual harassment and providing prompt treatment could be an effective deterrent.

*“One of the male nurses was sending me love messages and pornographic photos. I was silent at first, but the harassment continued, and I reported the hospital security guard. The guard called the nurse very quickly and warned him)” (Participant No. 13, a female nurse with 8 years of clinical work experience).*

## Discussion

This qualitative study aimed to identify strategies to prevent workplace sexual harassment among Iranian nurses. This study highlighted the basic measures to prevent and manage sexual behaviors in the workplace of nurses. According to the present study, nurses have proposed various strategies to prevent sexual harassment in the workplace. Four categories were extracted from nurses’ experiences: (1) behavioral response; (2) working conditions adjustment; (3) informing; and (4) the performance of hospital security guards.

### Behavioral response

Nurses who took part in the study revealed that one strategy for dealing with sexual harassment in the workplace was behavioral response. The study by Jenner et al. (2022) showed that preventive measures should be taken at both individual and institutional levels. Individual options include personal safety measures and personal protection strategies against patients, peers, and superiors (Jenner et al., 2022). Ross et al. (2019) advocated for mandatory policies that require healthcare organizations and nurses to model appropriate behaviors in order to reduce sexual harassment (Ross et al., 2019). The current study found that nurses could effectively prevent sexual harassment in the workplace by using appropriate behavioral responses, deterrent behaviors, and communications privacy. Some studies have suggested some communication strategies to control sexual harassment. When a patient harasses you, walk away and tell them that their behavior or treatment is unacceptable. Explain that you will not be able to continue their treatment/procedure unless their behavior changes and that you anticipate appropriate behavior (Ross et al., 2019). However, the type of behavior should be considered. While some behavior is so blatantly offensive that it is clearly sexual harassment, much of the behavior falls into a gray area. This behavior does not constitute sexual harassment if it is welcomed. Supervisors can date employees, and coworkers can laugh at dirty jokes, hug each other, and slap each other on the buttocks. However, if none of them objects, it is not sexual harassment (Lockwood, 2021). If, however, someone does object or is subjected passively to this behavior, it is sexual harassment. The prudent employer establishes a policy that precludes behavior that could be considered sexual harassment, and the prudent employee avoids all such behavior (NFA, 2016; Lockwood, 2021). The healthcare organization must create educational brochures that include information about the healthcare system’s expectations of patients as well as inappropriate behaviors (such as sexual harassment, disrespectful behavior, rudeness, or bullying) (Ross et al., 2019). However, because different societies have different social, cultural, and moral contexts, more research is needed to figure out what nurses should do in the workplace to stop sexual harassment.

### Working conditions adjustment

A review of the literature showed that workplace harassment adjustment has received less attention. Jenner et al.’s (2022) study found that organizational strategies include workplace guidelines and policies, structured grievance and reporting methods, formal training options, and organizational development and leadership strategies (Jenner et al., 2022). Adoption of appropriate policies in the workplace can create a safe workplace for nurses against sexual harassment (NFA, 2016), which is complex and common in the healthcare system because there are many medical institutions with different ward, supervisors, and different levels of authority (Lockwood, 2021). While sexual harassment by healthcare staff and patients is not acceptable, they often harass nurses because of this complex and multifaceted system. Specific working conditions expose nurses to sexual harassment (Zeighami et al., 2021), which are ward that increase the likelihood of sexual harassment. Patients in the emergency department may be confused or may be abusing drugs or alcohol, and as a result, they may occasionally make inappropriate remarks. Additionally, nurses should devise a strategy for preventing abusive behavior by patients with dementia who may not be aware of their actions (Ross et al., 2019). All of these factors may contribute to sexual harassment and other forms of harassment against nurses because of their unique work environment. Healthcare facilities should consider interventional policies and plans to address these conditions, and if none exists, nurses should request a specific and legally binding policy. Nurses should also discuss inappropriate behaviors and events with their colleagues and supervisors to help prevent sexual harassment. If these behaviors persist, they must adhere to organizational policies. Patient assignment, discharge, and coordination with hospital security are examples of such policies (Ross et al., 2019). It should be noted that the employer could not be held liable for conduct of which he is unaware. Employees must notify their employers if they are harassed (unless there is reason to fear for their personal safety). An employer can reprimand a harasser. If the harasser continues to harass a victim, the victim must report the harassment. If the victim fails to comply, the employer may not be held liable (Ross et al., 2019). Some studies have shown that a clear and consistent message from organizational leaders is essential. This is done through a written and widely circulated policy on sexual harassment. Organizations need to conduct regular self-assessments of sexual harassment and perceptions of the organizational climate. Adjusting working conditions is one of the most important ways to deal with sexual harassment among nurses. This is because different social, cultural, and moral contexts, as well as different hospital policies and management, can have a direct effect on preventing and reducing sexual harassment among nurses.

### Informing

Ross et al. (2019) advocated for a comprehensive action plan that included policies and staff training to empower nurses in healthcare organizations to prevent sexual harassment. Zeighami et al. (2021) demonstrated the importance of developing educational programs for nurses in order to enhance their coping skills. According to Kim et al. (2018), factors such as a lack of education, an inability to respond appropriately, and a nurse’s imbalance with patient commitments all contributed to nursing students’ experiences of sexual harassment in clinical settings. Tollstern Landin et al. (2020) conducted a study on sexual harassment among nurses and nursing students in Sub-Saharan Africa, demonstrating the need for increased knowledge about sexual harassment and the importance of sexual harassment education. Hospitals and medical schools should improve nurses’ knowledge and awareness of sexual harassment (Tollstern Landin et al., 2020). Sexual harassment prevention can be shared and discussed openly as part of a professional culture; nurses and caregivers must recognize, solve, and avoid their various work-related problems. Training can focus on finding a balance between the sexual needs of patients and the need to protect caregivers from bad things happening. It can also focus on finding a balance between professional distance and the chance of being affected by things that happen at work. Nursing students are recommended to identify and deal with sexual harassment effectively (Davis and Richardson, 2017). According to Birks et al. (2018), a sexual harassment prevention training program must be developed for nurses. Some studies have shown that regular training for all members of the organization, formal and informal reporting, research, and corrective action on sexual harassment are required (Buchanan et al., 2014). In addition to preventing sexual harassment, the training should also focus on how to deal with, report, and track sexual harassment to prevent complications following sexual harassment (Lockwood, 2021). Steps to prevent and report sexual harassment should be taken into account by healthcare organizations and managers. Education and knowledge of the rules are important parts of this process. However, more research is needed on the factors associated with sexual harassment and how to prevent it.

### Performance of hospital security guards

Nielsen et al. (2017) discovered that many nursing workplaces, including hospitals, nursing homes, social health centers, rehabilitation centers, and psychiatric residency centers, rarely followed instructions or policies to manage and/or prevent sexual harassment. According to Kim et al. (2018), when nursing students were sexually harassed during their clinical course, the harassers were not punished. As a result, violence management teams in the workplace must be established in order to enact rules and regulations that can ensure the safety of nurses in the workplace (Ali and Ezz El Rigal, 2019). Ali and Ezz El Rigal (2019) demonstrated that documenting any event and pursuing legal action, condemning sexual abuse, and strengthening harsh punishment for harassers were effective strategies for dealing with sexual harassment. Furthermore, the nurses in the current study relied on legal action against the harasser and the pursuit of sexual harassment reports. Healthcare facilities should make greater efforts to develop and implement strategies to reduce sexual harassment and create a safe work environment for nurses. Employers must protect their employees from sexual harassment. Sexual harassment must be addressed by the authorities, and it must be corrected as soon as possible (Lockwood, 2021). According to Nielsen et al. (2017), managers and safety agents were unaware of the frequency and impact of these events on caregivers. Developing and implementing coping strategies necessitates careful deliberation and reflection on the part of caregivers, managers, and safety agents. Patients’ sexual needs should be discussed as part of a professional culture, and various strategies to prevent and reduce the negative effects of sexual harassment should be identified (Nielsen et al., 2017). Nurse managers should put a lot of emphasis on how well law enforcement and sexual harassment protocols are used in healthcare settings, as well as how well nurses are helped to stop and reduce sexual harassment. As a way to stop sexual harassment, guards and other deterrents should also be thought about.

To deal with sexual harassment in the workplace, nurses need a variety of strategies, including high levels of knowledge and awareness (Tollstern Landin et al., 2020), identification of sexual harassment (Nielsen et al., 2017; Kim et al., 2018), early preparation (Davis and Richardson, 2017), self-care (Kim et al., 2018), correct reporting (Birks et al., 2018), prevention and management of sexual misconduct (Nielsen et al., 2017), security systems and policy formulation (Ali and Ezz El Rigal, 2019), and the use of effective coping strategies in nursing (Kim et al., 2018). Legal action, condemnation of sexual abuse, and stricter punishment for harassers are all policies that must be implemented in order to reduce harassment and create a safe working environment for nurses (Ali and Ezz El Rigal, 2019). Hospitals and medical schools should educate and raise the awareness of nursing students about sexual harassment (Tollstern Landin et al., 2020). They must assist nursing students in detecting and dealing with sexual harassment during clinical practice, which can improve nurses’ sensitivity (Lee et al., 2011) and prevent some future clinical issues. This necessitates a quick and decisive reporting system (Kim et al., 2018). To reduce sexual harassment in the workplace, violence management teams must be established, and appropriate rules and regulations must be enacted to improve workplace safety and patient care quality (Ali and Ezz El Rigal, 2019). Fundamental measures must be implemented to prevent and manage sexual harassment as a visible part of professional organizational culture (Nielsen et al., 2017). The study by Jenner et al. (2022) showed that the prevention of sexual harassment depends on a combination of individual and systemic measures to portray the personal and organizational dimensions of sexual harassment. A concerted effort to address both sides will sensitize the workforce, support victims, and prevent sexual harassment in medical institutions (Jenner et al., 2022). However, in the fight against sexual harassment in the workplace, social, cultural, and moral differences must be considered. Through this study, we hope to learn how nurses deal with sexual harassment in the workplace and to assist healthcare managers in creating a safe work environment for nurses.

### Limitations

Despite the fact that the confidentiality of the information and the findings of the interview were stressed, the interviewees may not have disclosed all of the sensitive information about sexual harassment. The findings should be generalized with caution because the majority of the participants in this study were women. According to research, the power imbalance between the perpetrator of sexual harassment and women, the patriarchal attitude of society, attention to women as a sexual means, lack of supervision in some areas of work such as the private sector, misconceptions toward nurses and women, and blaming them, are among the main reasons why most victims of sexual harassment in the workplace are women, and men have much less experience of sexual harassment. Due to the numerous cultural and ethnic differences in Iran and because the current study was conducted in southeastern Iran, it is necessary to pay attention to these differences in future studies. Future studies with quantitative and mix method design are suggested to address and confirm our findings in other cultures and settings. However, because of differences in the healthcare system and socio-cultural contexts, more research on a better understanding of strategies to prevent sexual harassment among nurses appears necessary. Since the data was collected during the COVID-19 disease crisis, its limitations should also be taken into account. In the future, studies should also look at how crises like COVID-19 affect sexual harassment.

## Conclusion

The findings of this study provide important and practical insights into perceptions of nurses’ coping strategies with sexual harassment in the workplace. This study showed that nurses proposed various experiences, such as behavioral response, working conditions adjustment, informing, and the performance of hospital security guards as strategies to prevent sexual harassment in the workplace. Nurse managers must take immediate and comprehensive measures to prevent and manage sexual misconduct against nurses. Healthcare managers and policymakers must design, develop, and promote practical guidelines for the legal accountability and safety of work environments to prevent sexual harassment. In addition, they must design educational, interventional, and prevention strategies and improve the coping skills of nurses. More quantitative and qualitative research needs to be done in other healthcare groups and in different parts of the world in order to understand how nurses deal with sexual harassment in the workplace.

## Data availability statement

The raw data supporting the conclusions of this article will be made available by the authors, without undue reservation.

## Ethics statement

The studies involving human participants were reviewed and approved by Kerman University of Medical Sciences. The patients/participants provided their written informed consent to participate in this study.

## Author contributions

MZe, PM, and MD designed the study and collected the data. MZe and MZa wrote the manuscript. MZe, MZa, PM, and MD contributed to the study design, provided critical feedback on the study and qualitative analysis, inputted to the draft of this manuscript, read, and approved the final manuscript.
